# Practical Performance Analysis of MDI-QKD with Orbital Angular Momentum on UAV Relay Platform

**DOI:** 10.3390/e26080635

**Published:** 2024-07-27

**Authors:** Dan Wu, Jiahao Li, Lan Yang, Zhifeng Deng, Jie Tang, Yuexiang Cao, Ying Liu, Haoran Hu, Ya Wang, Huicun Yu, Jiahua Wei, Huazhi Lun, Xingyu Wang, Lei Shi

**Affiliations:** 1 Information and Navigation College, Air Force Engineering University, Xi’an 710077, China; yay1hh@163.com (D.W.); quantum_ljh@163.com (J.L.); dzf1219847064@163.com (Z.D.); 18014102582@163.com (J.T.); cyxdm2021@163.com (Y.C.); 15030433826@163.com (Y.L.); huhaoran02061022@163.com (H.H.); wangya98@126.com (Y.W.); yhc9426@163.com (H.Y.); weijiahua@126.com (J.W.); huazhilun@tom.com (H.L.); 2 National Key Laboratory of Complex Aviation System Simulation, Beijing 100101, China; 13651231371@126.com

**Keywords:** orbital angular momentum, MDI-QKD, unmanned aerial vehicles, atmospheric turbulence, state-dependent diffraction, weather visibility

## Abstract

The integration of terrestrial- and satellite-based quantum key distribution (QKD) experiments has markedly advanced global-scale quantum networks, showcasing the growing maturity of quantum technologies. Notably, the use of unmanned aerial vehicles (UAVs) as relay nodes has emerged as a promising method to overcome the inherent limitations of fiber-based and low-Earth orbit (LEO) satellite connections. This paper introduces a protocol for measurement-device-independent QKD (MDI-QKD) using photon orbital angular momentum (OAM) encoding, with UAVs as relay platforms. Leveraging UAV mobility, the protocol establishes a secure and efficient link, mitigating threats from untrusted UAVs. Photon OAM encoding addresses reference frame alignment issues exacerbated by UAV jitter. A comprehensive analysis of atmospheric turbulence, state-dependent diffraction (SDD), weather visibility, and pointing errors on free-space OAM-state transmission systems was conducted. This analysis elucidates the relationship between the key generation rate and propagation distance for the proposed protocol. Results indicate that considering SDD significantly decreases the key rate, halving previous data results. Furthermore, the study identifies a maximum channel loss capacity of 26 dB for the UAV relay platform. This result is pivotal in setting realistic parameters for the deployment of UAV-based quantum communications and lays the foundation for practical implementation strategies in the field.

## 1. Introduction

As one of the first applications of quantum mechanics, quantum key distribution (QKD) [1,2] has facilitated the emergence of quantum information technology and laid the foundation for the establishment of quantum networks [3,4]. In recent years, with the breakthroughs in metropolitan [5] and satellite–ground [6,7] quantum communication networks, a global quantum internet has become a grand goal which drives countries to carry out relevant research. However, the transmission limitation of optical fibers and the fixed trajectory of satellites restrict the flexibility and scalability of quantum communication networks. Inspired by pioneering demonstrations of drone-based entanglement distribution, the unmanned aerial vehicle (UAV) platform [8,9], based on its mobility and maneuverability, has shown potential as relay nodes for free-space QKD. In complex environments where optical fibers cannot be laid, such as mountains, islands, and urban high-rise buildings that satellites cannot cover, UAVs can be easily moved and positioned [10,11], enabling the flexible formation of UAV quantum networks. Additionally, they can achieve the information interconnection between satellite-based and fiber-based quantum networks, which serve as a critical supplement targeting the integrated quantum communication network.

In the practical implementation of QKD systems, significant security vulnerabilities exist that cause quantum communication processes to fail. In 2012, Lo et al. proposed the measurement-device-independent QKD (MDI-QKD) protocol [12], which addressed the security risks at the detection end and showed advantages in extending propagation distances [13]. Therefore, the potential security issues in UAVs can be effectively mitigated by MDI-QKD. However, existing implementations of MDI-QKD are affected by basis-dependent flaws [14]. Reference-frame-independent QKD (RFI-QKD) [15,16] and orbital angular momentum QKD (OAM-QKD) [17,18] have been demonstrated to address frame alignment issues. According to the transmission characteristics of OAM in free space, UAV quantum communication can serve as a supplement to integrate quantum communication networks. In contrast to the fixed motion trajectory of satellite scenarios more suitable to RFI-QKD, the performance of UAV scenarios is constrained by a reference frame caused by the high-speed mobility and random attitude of UAV platforms. Thus, OAM-QKD is considered a promising selection for UAV scenarios with its rotational invariance. Furthermore, the OAM degree of freedom theoretically offers an infinite-dimensional encoding space, and high-dimensional OAM-QKD can improve channel robustness and increase information encoding capacity [19], opening up new possibilities for high-capacity UAV-based quantum communications.

Recent research has laid the foundation for the preliminary exploration of OAM-QKD in free space, including demonstration experiments of OAM-encoded QKD links within urban areas [20,21], a feasibility analysis of OAM-QKD from satellites to the ground [22], model crosstalk studying between adjacent OAM modes [23], adaptive optics (AO) compensation for wavefront aberrations [24], and the optimization of optical wavelengths and filtering methods [25]. However, UAV-based quantum channels face unique challenges in free space, and most existing work has not fully considered the effects of beam diffraction, atmospheric scattering, turbulence, weather visibility and pointing jitter of UAVs. Therefore, the theoretical feasibility of achieving OAM-MDI-QKD based on UAV platforms with limited receiving apertures remains ambiguous. To bridge the gap between theory and practice, this paper uses a three-intensity decoy-state protocol [26] and finite-key security [27] analysis to present the relationship between the key rate and propagation distance for the OAM-encoded MDI-QKD protocol. Additionally, we analyzed and simulated the potential challenges faced by UAV-based OAM-MDI-QKD links, including diffraction, turbulence, weather visibility, and pointing error, providing new ideas for UAV-based quantum secure communication.

This paper first proposes the channel model for the UAV-based OAM-MDI-QKD in Section 2, studying the theoretical derivation of the diffraction, turbulence, and weather visibility of an OAM beam in free space. Additionally, a brief analysis is conducted on the effects of UAV jitters. In Section 3, we present the key rate analysis of the OAM-MDI-QKD protocol combined with the three-intensity decoy-state and finite-key analysis. In Section 4, we discuss the impact of channel challenges under different simulation settings and comprehensively consider the performance of the system key rate under diffraction, turbulence, weather visibility, and pointing error. The conclusion of this paper is given in Section 5.

## 2. Ground-To-Uav Vortex Beam Propagation Model for UAV-Based MDI-QKD

**Figure 1:**
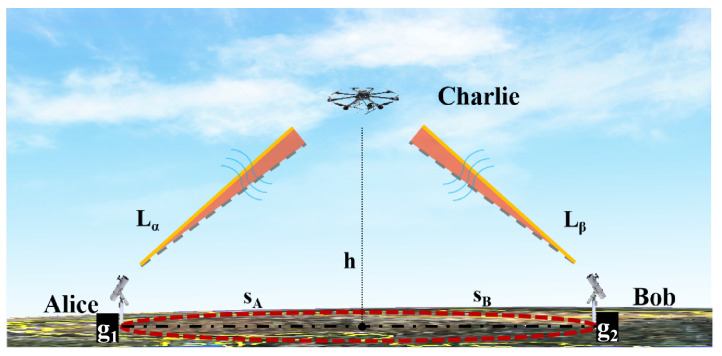
Schematic diagram of ground-to-UAV MDI-QKD scenarios.

We here consider a general application of UAV-based MDI-QKD between two ground stations. As shown in Figure 1, Alice and Bob are the MDI-QKD photon sources that randomly and individually prepares one of four BB84 states using the phase-randomized weak coherent pulse (WCP) sources with decoy signals. Then they transmit the OAM-encoded quantum signals to an untrusted third party, which performs the Bell state measurement (BSM) on a UAV and announces the results through a public radio channel. For simplicity, Alice and Bob are located at ground stations $g_{1}$ and $g_{2}$, respectively, which are separated by a distance $S=S_{A}=S_{B}$ with a UAV at altitude h at the midpoint. What makes UAV applications difficult are that the adverse effects, such as geometric spread, atmospheric turbulence, weather dependence, pointing error, etc., in practice, incur very high attenuation which results in key rate degradation of the UAV-based MDI-QKD. In particular, for vortex beams, all of the above factors have more severe effects on OAM-based MDI-QKD, which will be discussed in the following.

### 2.1. State-Dependent Diffraction of OAM Considerations

**Figure 2:**
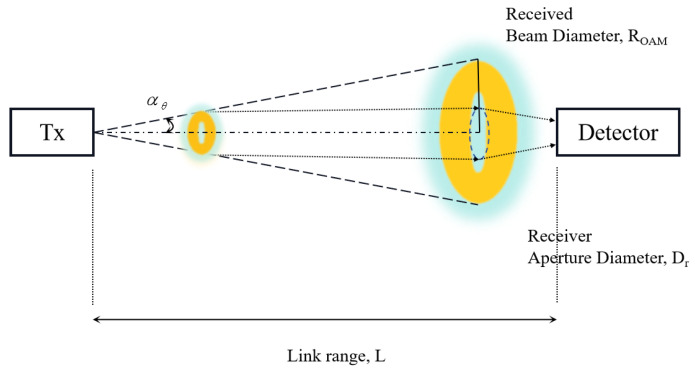
Schematic diagram of state-dependent diffraction.

**Figure 3:**
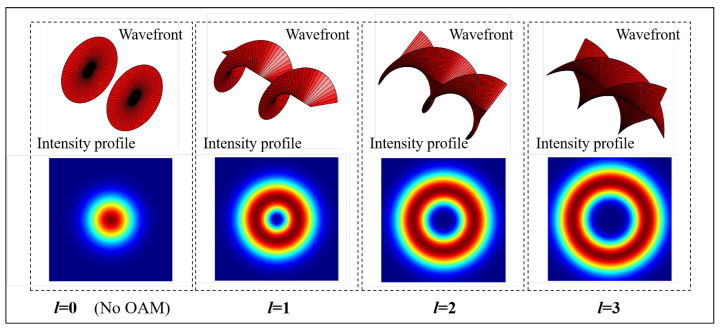
The wavefronts and intensity profiles of OAM modes l=0,1,2, and 3. The OAM mode with a nonzero order has a donut-shaped intensity profile. The size of the ring in the intensity profile grows with *l*.

When performing uplink MDI-QKD to a UAV that typically cannot be equipped with a large receiving telescope because of the aerodynamic structure and load limitations, the geometric loss contributes significantly. This occurs due to the beam diffraction, which scales inversely with the square of the propagation distance, with the final beam width potentially being larger than the diameter of the receiving telescope. As illustrated in Figure 2, beam diffraction induces a gradual spread of the optical beam with increasing propagation distance, potentially causing incomplete signal reception at the receiver and resulting in beam divergence loss. As shown in Figure 3, a vortex beam brings up even more broadening than the Gaussian beam, because of its donut-shaped intensity profiles with a null at the center. Additionally, the state-dependent diffraction (SDD) of OAM creates the detection efficiency mismatch for receiving the different-order OAM states [20], which leads to the side-channel vulnerabilities [28]. In our UAV-based application, the MDI-QKD protocol is employed to counter it, and a decoy-state technique is used to combine transmittances for different OAM states, which is discussed in the following.

First, in this section, we analyzed the influence of SDD on the application scenario and utilized the intensity distribution of the vortex beam from [29], given by

(1) $$\begin{array}{c} I_{I}\left(r,\phi ,z\right)=\frac{2}{w{\left(z\right)}^{2}\pi |l|!}{\left(\frac{\sqrt{2}r}{w(z)}\right)}^{2|l|}\exp\left(-\frac{2r^{2}}{w{\left(z\right)}^{2}}\right), \end{array}$$

where *l* is the order of the OAM state, which represents the number of 2π phase shifts in the azimuthal direction order. w(z) is the beam radius at the receiving plane *z*, which is given as

(2) $$w\left(z\right)=w_{0}\sqrt{1+L^{2}/z_{R}^{2}},$$

where *L* is the propagation distance, $w_{0}$ is the beam radius, and $z_{R}$ can be expressed as

(3) $$z_{R}=\frac{1}{2}kw_{0}^{2}.$$

Here, λ is the laser wavelength and k=2π/λ is the wavelength. We consider that the receiving telescope can be described as a circular aperture of diameter $D_{r}$, which collects part of the incoming beam and focuses it on a bucket single-photon detector. Combing Equation (1) with Equations (2) and (3), the power $P_{0}$ received through a circular aperture of diameter $D_{r}$ centered on the vortex beam is

(4) $$P_{0}=\int_{0}^{\frac{D_{r}}{2}}I_{s}\left(r,\phi ,z\right)dr.$$

We assume that the transmission power *P* centered on the vortex beam is 1; therefore, the link efficiency $\eta_{SDD}$, which we define as the percentage of the received power with respect to the transmitted one, is

(5) $$\eta_{SDD}=P_{0}/P.$$

In addition, the variations in attitude of the drone platform cause a misalignment with the ground station, leading to a significant decrease in the reception efficiency of the finite-aperture telescope following beam diffraction. Therefore, it is crucial to address the losses in pointing accuracy resulting from UAV jitter. The error attributed to misalignment is given as [30]

(6) $$G\left(\theta_{j}\right)=\exp\left(-8\theta_{j}^{2}\left(\alpha_{\theta }^{-2}\right)\right),$$

where $\theta_{j}$ is the beam jitter angle, and $\alpha_{\theta }$ is the transmitter beam divergence. Thus, the protocol transmission probability, affected solely by pointing error loss, is expressed as

(7) $$\eta_{P}=\exp\left[-G\left(\theta_{j}\right)L\right].$$

### 2.2. Atmospheric Turbulence of OAM Considerations

In a turbulent channel, continuous random fluctuations manifest. As a light beam traverses through such turbulent media, the random fluctuations of the medium induce random phase fluctuations in the light beam, introducing perturbations to the beam’s propagation path. Consequently, the transmitted orbital angular momentum (OAM) state scatters from its original mode to adjacent OAM modes, giving rise to OAM mode crosstalk. This phenomenon contributes to a reduction in the secure key rate of the protocol system.

In this paper, we employed the Kolmogorov model to quantitatively assess the influence of atmospheric turbulence on the crosstalk of OAM states. Following passage through atmospheric turbulence, the receiver has a probability of receiving adjacent OAM states [31], denoted as $P_{\Delta l}$:

(8) $$P_{\Delta l}=\frac{1}{\pi }\int_{0}^{1}d\rho \rho \int_{0}^{2\pi }d\theta e^{-3.44{\left(D_{r}/r_{0}\right)}^{5/3}{\left(\rho \sin\theta /2\right)}^{5/2}}\cos\Delta l\theta ,$$

where $\rho =r/R_{r}$, *r* is the radial coordinates, $R_{r}$ and $D_{r}$ are the diameter and radius of the receiver aperture, and $r_{0}$ is the Fried parameter that describes the effective coherence length of turbulence:

(9) $$r_{0}=0.1853{\left(\frac{\lambda^{2}}{C_{n}^{2}L}\right)}^{3/5},$$

where $C_{n}^{2}$ is the refractive-index structure parameter and λ is the wavelength. When Δl=0, it denotes the probability of receiving OAM states consistent with the transmitted ones.

In the OAM-MDI-QKD system, the transmission probability $\eta_{0}$ is the probability of correctly transmitting the OAM state, which represents the probability of receiving photons with the same OAM state as the transmitted one; the transmission probability $\bar{\eta_{0}}$ is the probability of erroneously transmitting the OAM state, which represents the probability of receiving photons with a different OAM state from the transmitted ones. Thus, $\eta_{0}$ and $\bar{\eta_{0}}$ can be expressed as

(10) $$\begin{array}{c} \eta_{0}=\frac{e^{-\beta L}}{\pi }\int_{0}^{1}\rho d\rho \times \int_{0}^{2\pi }\exp\left[-3.44{\left(\frac{D_{r}}{r_{0}}\rho \sin\left(\frac{\theta }{2}\right)\right)}^{5/3}\right]d\theta \\ \bar{\eta_{0}}=\frac{e^{-\beta L}}{\pi }\int_{0}^{1}\rho d\rho \times \int_{0}^{2\pi }\exp\left[-3.44{\left(\frac{D_{r}}{r_{0}}\rho \sin\left(\frac{\theta }{2}\right)\right)}^{5/3}\right]\cos\left(2l\theta \right)d\theta , \end{array}$$

where β is the link attenuation coefficient due to atmospheric absorption and scattering. Therefore, the protocol transmission probability under the influence of atmospheric turbulence and link attenuation is given as

(11) $$\eta_{a}=\eta_{0}+\bar{\eta_{0}}.$$

### 2.3. Weather Visibility of OAM Considerations

The performance of OAM-encoded optical signals propagated through free space is subject to weather conditions like fog, rain, snow, etc., that lead to a decrease in the received signal power. Among these conditions, fog typically exerts a more substantial impact on atmospheric attenuation due to the particle size of fog. Fog has the capability to modify optical signal characteristics and impede light transmission comprehensively through processes that encompass absorption, scattering, and reflection. Mie, employing electromagnetic theory, formulated theoretical expressions for scattering efficiency [32]. When the scattering particle size distribution is provided, the total scattering or attenuation coefficient α can be determined by summing contributions from individual particle sizes [33]:

(12) $$\alpha =\sum_{i}\xi_{i}\sigma_{i}\pi r_{i}^{2},$$

where $\xi_{i}$ is distribution or concentration of the ith particle, $\sigma_{i}$ is scattering efficiency of the of the ith particle, and $r_{i}$ is radius of the ith particle. Unfortunately, the scattering particle size distribution is not immediately accessible, making the utilization of Equation (12) for determining the attenuation coefficient often impractical. However, an alternative form of Equation (12) has been developed, relying solely on the more commonly available parameter—visibility. Assessing atmospheric visibility emerges as a valuable method to anticipate and appraise atmospheric environmental conditions. This form to compute the atmospheric attenuation coefficient is convenient, as the attenuation is solely contingent on visibility for a given wavelength. Visibility, a readily accessible parameter, can be obtained from airport or weather data. Reference [30] presents empirical models focusing on visibility-based attenuation. For our computations, we have chosen to employ the Kim model, which provides a representation of atmospheric attenuation:

(13) $$U\left(\lambda \right)=\frac{3.91}{V}{\left(\frac{\lambda }{550}\right)}^{-s},$$

where $U\left(\lambda \right)$ is the atmospheric attenuation coefficient, V(km) is the weather visibility range, and *s* is the scattering particle size distribution, defined by:

(14) $$s=\left\{\begin{array}{cc} 1.6 & V>50\\ 1.3 & 6<V<50\\ 0.16V+0.34 & 1<V<6\\ V-0.5 & 0.5<V<1\\ 0 & V<0.5 \end{array}\right..$$

Therefore, the protocol transmission probability, affected solely by weather visibility, is expressed as

(15) $$\eta_{V}=\exp\left[-U(\lambda )L\right].$$

## 3. Protocol and Key Rate

Based on the established link model in this paper, we employed two mutually unbiased bases for encoding optical signals, the OAM basis denoted as

(16) $$|\Psi_{\mathrm{OAM}}^{n=0}\rangle =|-l\rangle ,|\Psi_{\mathrm{OAM}}^{n=1}\rangle =|l\rangle ,$$

and the SUP basis denoted as

(17) $$|\Psi_{\sup}^{n}\rangle =\frac{1}{\sqrt{2}}(|l\rangle +e^{isn\pi }|-l\rangle ),\left(n=0,1\right).$$

**Figure 4:**
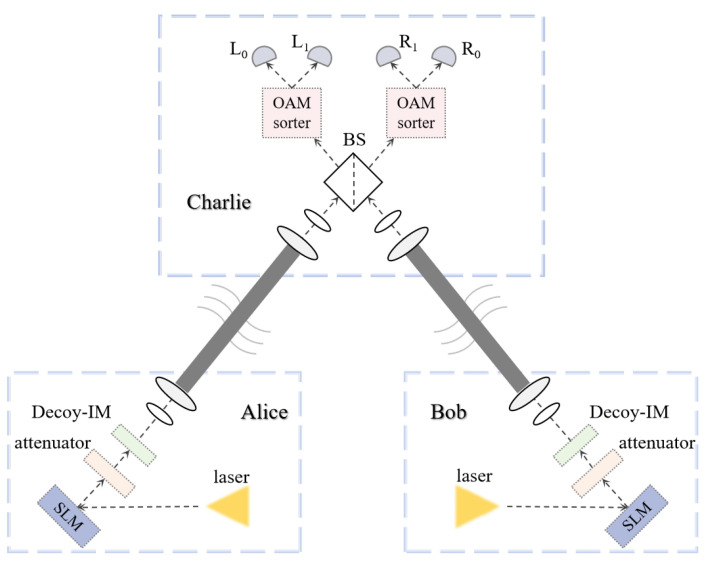
Schematic diagram of OAM-MDI-QKD.

Due to the superposition nature of the SUP basis, its two states lack rotational invariance, requiring consideration for aligning the shared reference frame. In Figure 4, Alice and Bob independently and randomly choose between encoding information using either the OAM or SUP basis. A spatial light modulator (SLM) is utilized to modulate the required OAM states, followed by attenuation to obtain weak coherent states. These states are then modulated into the desired signal and decoy states using a Decoy-IM. In the protocol, three intensity decoy states are employed: the vacuum state, the decoy state, and the signal state. These states are transmitted through the free-space turbulent channel to the third-party drone platform, Charlie. Within the UAV measurement apparatus, photons from Alice and Bob simultaneously reach a 50:50 beam splitter (BS) [34], interfering before being distinguished into different OAM states using a highly efficient OAM sorter [35,36]. Subsequently, detector responses are obtained. Charlie declares a successful measurement only when Alice and Bob both choose the OAM basis, whereas the results for the SUP basis are used to test protocol security.

In our protocol, the key generation rate [12] for Alice and Bob can be written as follows:

(18) $$R=\mu_{2}\nu_{2}\exp\left(-\mu_{2}-\nu_{2}\right)\underline{Y_{11}^{OAM}}\left(1-H\left(\bar{e_{11}^{SUP}}\right)\right)-Q_{\mu_{2}\nu_{2}}^{OAM}fH\left(E_{\mu_{2}\nu_{2}}^{OAM}\right),$$

where $Y_{11}^{OAM}$ is the gain when both Alice and Bob select the OAM basis and send single-photon states, and $\underline{Y_{11}^{OAM}}$ represents the lower bound of $Y_{11}^{OAM}$. $e_{11}^{\mathrm{SUP}}$ is the quantum bit error rate (QBER) when both Alice and Bob select the SUP basis and send single-photon states. $Q_{\mu_{2}\nu_{2}}^{OAM}$ stands for the total gain when Alice and Bob both choose the OAM basis to send signal states, while $E_{\mu_{2}\nu_{2}}^{OAM}$ is the total QBER in this scenario. Additionally, $H\left(x\right)=-x\log_{2}\left(x\right)-\left(1-x\right)\log_{2}\left(1-x\right)$ is the binary Shannon entropy function, and *f* is the system error correction efficiency. While employing an ideal infinite set of decoy states would yield highly accurate values for $Y_{11}^{OAM}$ and $e_{11}^{\mathrm{SUP}}$, practical limitations, such as finite-source capabilities and finite-key lengths, necessitate the consideration of statistical fluctuations with finite-length keys. Thus, we adopted a three-intensity decoy state approach to separately estimate the lower bound $\underline{Y_{11}^{OAM}}$ and $\bar{e_{11}^{\mathrm{SUP}}}$, the upper bound, under conditions of finite-key length. We define the pulse strengths of Alice and Bob as $\left\{\mu_{i}\right\},i=0,1,2,$ and $\left\{\nu_{j}\right\},j=0,1,2$, corresponding to the vacuum state, decoy state, and signal state, respectively. The gain $Q_{\mu_{i}\nu_{j}}^{w}$ and error rate $E_{\mu_{i}\nu_{j}}^{w}$ are given by

(19) $$Q_{\mu_{i}\nu_{j}}^{w}=\sum_{n,m=0}^{\infty }\frac{\mu_{i}^{n}\nu_{j}^{m}}{nm!}e^{-\mu_{i}-\nu_{i}}Y_{nm}^{w},$$

(20) $$E_{\mu_{i}\nu_{j}}^{w}Q_{\mu_{i}\nu_{j}}^{w}=\sum_{n,m=0}^{\infty }\frac{\mu_{i}^{n}\nu_{j}^{m}}{nm!}e^{-\mu_{i}-\nu_{i}}Y_{nm}^{w}e_{nm}^{w},$$

where *w* is OAM or SUP bases, *m* and *n* represent the number of photons that Alice and Bob send in pulses, respectively. According to the statistical fluctuation analysis proposed in Ref. [37], the calculation method of total gain $Q_{\mu_{i}\nu_{j}}^{w}$ and $E_{\mu_{i}\nu_{j}}^{w}$ can be further limited to

(21) $$Q_{\mu_{i}\nu_{j}}^{w}\left(1-\delta_{q}\right)⩽Q_{\mu_{i}\nu_{i}}^{w}⩽Q_{\mu_{i}\nu_{j}}^{w}\left(1+\delta_{q}\right),$$

(22) $$E_{\mu_{i}\nu_{j}}^{w}Q_{\mu_{i}\nu_{j}}^{w}\left(1-\delta_{eq}\right)⩽E_{\mu_{i}\nu_{j}}^{w}Q_{\mu_{i}\nu_{i}}^{w}⩽E_{\mu_{i}\nu_{j}}^{w}Q_{\mu_{i}\nu_{j}}^{w}\left(1+\delta_{eq}\right).$$

In which

(23) $$\delta_{q}=\frac{n_{\alpha }}{\sqrt{N_{\mu_{i}\nu_{j}}^{w}\bar{Q_{\mu_{i}\nu_{j}}^{w}}}},\delta_{eq}=\frac{n_{\alpha }}{\sqrt{N_{\mu_{i}\nu_{j}}^{w}\bar{E_{\mu_{i}\nu_{j}}^{w}}\bar{Q_{\mu_{i}\nu_{j}}^{w}}}},$$

where $N_{\mu_{i}\nu_{j}}^{w}$ is the number of pulses based on *w* when Alice and Bob use signal strengths $\mu_{i}$ and $\nu_{j}$, respectively, and $n_{a}$ is the value of the standard deviation used for statistical fluctuation analysis. Thus, the lower bound for single-photon gain and the upper bound for single-photon QBER are given by

(24) $$Y_{11}^{x}⩾\underline{Y_{11}^{x}}=\frac{\underline{{g_{1}}^{x}}+\underline{{g_{2}}^{x}}+\underline{{g_{3}}^{x}}e^{\mu_{2}+\nu_{2}}\bar{Q_{\mu_{2}\nu_{2}}^{x}}+e^{\mu_{1}+\nu_{1}}\underline{Q_{\mu_{1}\nu_{1}}^{x}}}{\kappa \mu_{2}\nu_{1}+\kappa \mu_{1}\nu_{2}-\mu_{1}\nu_{1}-\mu_{2}\nu_{2}},$$

(25) $$e_{11}^{x}⩽\bar{e_{11}^{x}}=\frac{e^{\mu_{1}+\nu_{1}}\bar{Q_{\mu_{1}\nu_{1}}^{x}E_{\mu_{1}\nu_{1}}^{x}}-\underline{g_{4}^{x}}}{\mu_{1}\nu_{1}\underline{Y_{11}^{x}}},$$

In which

(26) $$\begin{array}{c} g_{1}^{x}=e^{\nu_{2}}Q_{0\nu_{2}}^{x}+e^{\mu_{2}}Q_{\mu_{2}0}^{x}-e^{\nu_{1}}Q_{0\nu_{1}}^{x}-e^{\mu_{1}}Q_{\mu_{1}0}^{x},\\ g_{2}^{x}=\kappa \left(e^{\mu_{2}+\nu_{1}}Q_{\mu_{2}\nu_{1}}^{x}-e^{\nu_{1}}Q_{0\nu_{1}}^{x}-e^{\mu_{2}}Q_{\mu_{2}0}^{x}+Q_{00}^{x}\right),\\ g_{3}^{x}=\kappa \left(e^{\mu_{1}+\nu_{2}}Q_{\mu_{1}\nu_{2}}^{x}-e^{\nu_{2}}Q_{0\nu_{2}}^{x}-e^{\mu_{1}}Q_{\mu_{1}0}^{x}+Q_{00}^{x}\right),\\ g_{4}^{x}=e^{\nu_{1}}Q_{0\nu_{1}}^{x}E_{0\nu_{1}}^{x}+e^{\mu_{1}}Q_{\mu_{1}0}^{x}E_{\mu_{1}0}^{x}-Q_{00}^{x}E_{00}^{x}, \end{array}$$

(27) $$\kappa =\min\left(\frac{\mu_{2}\nu_{2}^{2}-\mu_{1}\nu_{1}^{2}}{\mu_{2}\nu_{1}^{2}+\mu_{1}\nu_{2}^{2}},\frac{\mu_{2}^{2}\nu_{2}^{2}-\mu_{1}^{2}\nu_{1}^{2}}{\mu_{2}^{2}\nu_{1}^{2}+\mu_{1}^{2}\nu_{2}^{2}},\frac{\mu_{2}^{2}\nu_{2}-\mu_{1}^{2}\nu_{1}}{\mu_{2}^{2}\nu_{1}+\mu_{1}^{2}\nu_{2}}\right),$$

Based on the formulae, we can derive the gains and QBERs when Alice and Bob send OAM states: when Alice and Bob both choose the SUP basis, the gains and QBER are denoted as follows:

(28) $$Q_{\mu_{i}\nu_{j}}^{SUP}=2y_{ij}^{2}\left[1+2y_{ij}^{2}-4y_{ij}I_{0}\left(s_{ij}\right)+I_{0}\left(2s_{ij}\right)\right],$$

(29) $$E_{\mu_{i}\nu_{j}}^{SUP}Q_{\mu_{i}\nu_{j}}^{SUP}=e_{0}Q_{\mu_{i}\nu_{j}}^{SUP}-2\left(e_{0}-e_{d}\right)y_{ij}^{2}\times \left[I_{0}\left(2s_{ij}\right)-1\right],$$

when Alice and Bob both choose the OAM basis, the gain is denoted as follows:

(30) $$Q_{\mu_{i}\nu_{j}}^{OAM}=\frac{1}{2}\left(Q_{\mu_{i}\nu_{j}}^{OAM_{C}}+Q_{\mu_{i}\nu_{j}}^{OAM_{E}}\right),$$

(31) $$\begin{array}{c} \begin{array}{c} Q_{\mu_{i}\nu_{j}}^{\mathrm{OAM}}C=4{\left(1-P_{d}\right)}^{2}e^{-\mu_{ij}^{\prime }/2}\left[1-\left(1-P_{d}\right)e^{-\mu_{i}\eta_{A}/2}\right]\left[1-\left(1-P_{d}\right)e^{-\nu_{j}\eta_{B}/2}\right], \end{array} \end{array}$$

(32) $$\begin{array}{c} \begin{array}{c} Q_{\mu_{i}\nu_{j}}^{\mathrm{OAM}}E=4{\left(1-P_{d}\right)}^{2}e^{-\mu_{ij}^{\prime }/2}\left[I_{0}\left(s_{ij}\right)-\left(1-P_{d}\right)e^{-\mu_{ij}^{\prime }}/2\right]. \end{array} \end{array}$$

In which

(33) $$\begin{array}{c} \begin{array}{c} \mu_{ij}^{\prime }=\eta_{A}\mu_{i}+\eta_{B}\nu_{j},\\ s_{ij}=\sqrt{\eta_{A}\mu_{i}\eta_{B}\nu_{j}}/2,\\ y_{ij}=\left(1-P_{d}\right)e^{\mu_{ij}^{\prime }/4},\\ \eta_{A}=\eta_{\mathrm{SDD}}\times \eta_{a}\times \eta_{V}\times \eta_{P}. \end{array} \end{array}$$

where $P_{d}$ is the single-photon detector dark count rate, $I_{0}\left(s_{ij}\right)$ is the first kind of modified Bessel function, and $\eta_{A}\left(\eta_{B}\right)$ is the transmittance of Alice (Bob). Since OAM states exhibit rotational invariance, the necessity for addressing alignment issues with a shared reference frame is obviated. Consequently, during communication in a free-space link, the QBER is exclusively influenced by atmospheric turbulence, beam diffraction, and weather visibility interference, expressed as

(34) $$E_{\mu_{i}\nu_{j}}^{\mathrm{OAM}}=\frac{\left(t_{a}\left(1-t_{b}\right)+\left(1-t_{a}\right)t_{b}\right)Q_{\mu_{i}\nu_{j}}^{{\mathrm{OAM}}_{C}}+\left(\left(1-t_{a}\right)\left(1-t_{b}\right)+t_{a}t_{b}\right)Q_{\mu_{i}\nu_{j}}^{{\mathrm{OAM}}_{E}}}{2Q_{\mu_{i}\nu_{j}}^{\mathrm{OAM}}},$$

where $t_{a}$ is the crosstalk probability in the link from Alice to drone Charlie, and $t_{b}$ is the crosstalk probability in the link from Bob to drone Charlie. Additionally, we make the assumption that $t_{a}=t_{b}$.

## 4. Performance Simulation

In this section, we conducted a performance analysis of our protocol through numerical simulations. The numerical parameters are detailed in Table 1.

**Figure 5:**
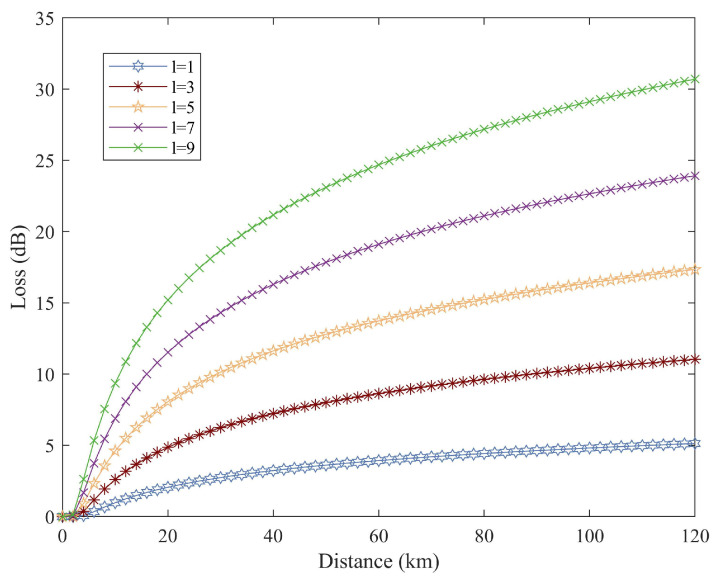
Link loss against propagation distance of different OAM orders l=1,3,5,7, and 9.

Figure 5 examines the influence of geometric losses on the transmission of OAM states. The results suggest that, under specified beam dimensions and fixed receiving apertures, state-dependent diffraction losses are contingent on the order *l* of the OAM state. With increasing distance, beam diffraction has a progressively more substantial impact on free-space transmission systems, disproportionately affecting higher-order OAM states.

**Figure 6:**
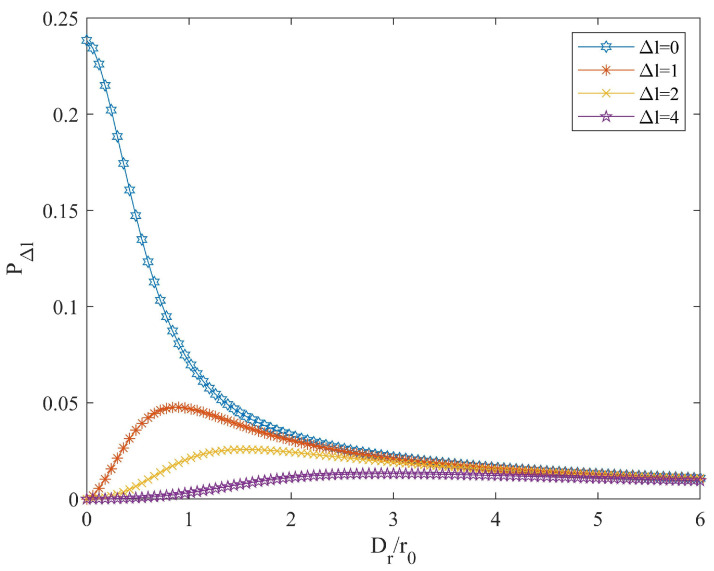
Probability of receiving adjacent OAM modes versus the ratio of the telescope diameter $D_{r}$ to the Fried parameter $r_{0}$.

To address the atmospheric turbulence effects discussed in Section 2.2, the probabilities of receiving adjacent modes are quantitatively calculated by using the Kolmogorov model, as depicted in Figure 6. The effect of receiving devices is quantified with $D_{r}/r_{0}$, where $D_{r}$ represents the diameter of the receiving telescope. When the receiving aperture $D_{r}$ is smaller, the reception of adjacent modes is incomplete; thus, the probabilities of receiving adjacent modes (Δl=1,2,4) increase and relatively reduce the probability of receiving the initial OAM state (Δl=0). As the aperture $D_{r}$ increases, the telescope receives a more complete beam, leading to a more complete extraction of OAM states. Consequently, the reception probability of adjacent OAM states (Δl=1,2,4) increases, thereby relatively decreasing the reception probability of the original OAM state.Ultimately, the OAM states tend towards random distribution, and the acceptance probabilities for each mode approach stability with similar numerical values.

**Figure 7:**
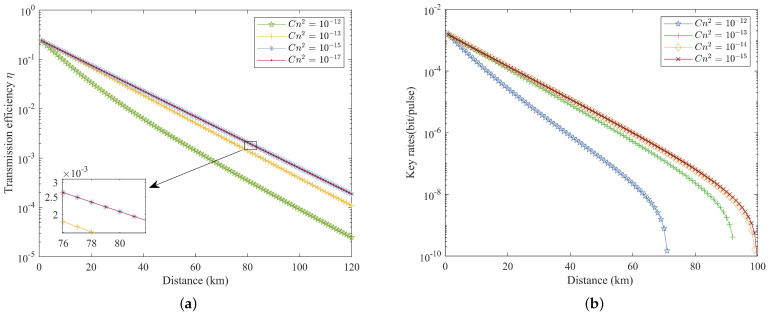
(**a**) Transmission efficiency against distance at different atmospheric turbulence strengths. (**b**) Key rate versus distance at different atmospheric turbulence strengths.

Typically, the strength of atmospheric turbulence is represented as $C_{n}^{2}$. When $C_{n}^{2}=10^{-17}m^{-2/3}$ or lower, turbulence is considered weak, while turbulence is deemed strong when $C_{n}^{2}=10^{-15}m^{-2/3}$ or higher. Figure 7a subsequently illustrates the correlation among different atmospheric turbulence strengths, propagation distance, and transmission efficiency, while Figure 7b delineates the relationship between propagation distance and the key rate of the protocol. These figures demonstrate that increased turbulence significantly reduces the achievable propagation distance of the protocol. Moreover, with escalating turbulence, the transmission efficiency, influenced solely by atmospheric turbulence, decreases, resulting in diminished key rates and shorter propagation distances. From the graph, it can be observed that the difference in transmission efficiency of the system is not significant at atmospheric turbulence strengths $C_{n}^{2}=10^{-17}m^{-2/3}$ and $C_{n}^{2}=10^{-15}m^{-2/3}$. The transmission quality is relatively good; hence, we often utilize this value in numerical simulations.

**Figure 8:**
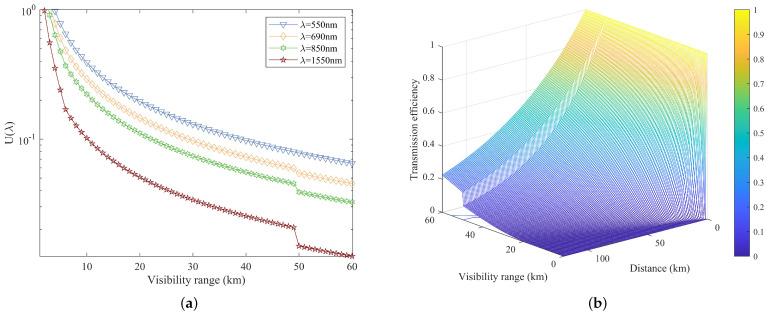
(**a**) Weather visibility versus attenuation at different wavelengths λ = 550 nm, 690 nm, 850 nm, and 1550 nm. (**b**) Relationship between weather visibility, propagation distance, and transmission efficiency at the operational wavelength of 1550 nm.

For a given wavelength, the attenuation value is exclusively dependent on weather visibility. At the operational wavelength of 550 nm, attenuation is relatively pronounced, gradually diminishing as the visibility range *V* increases. Conversely, for wavelengths exceeding 550 nm, particularly at λ=1550 nm, attenuation experiences a sharp decline when visibility equals or exceeds 50 km, consistent with the piecewise function representation of Equation (14). As illustrated in Figure 8a, weather visibility has the least impact on the propagation of OAM-encoded optical beams at the operational wavelength of 1550 nm compared to others. Figure 8b visually represents the relationship between weather visibility, propagation distance, and transmission efficiency at the operational wavelength of 1550 nm. It becomes evident that, due to the influence of weather, the impact on transmission efficiency becomes more pronounced with increasing propagation distance.

**Figure 9:**
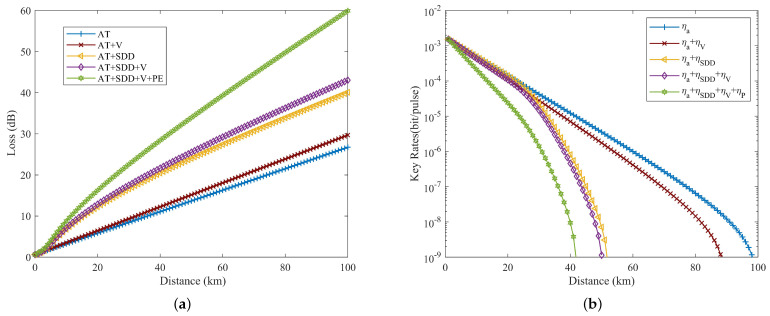
(**a**) Variation of the link loss as a function of the propagation distance for different effects. (**b**) Variation of the key rate as a function of the propagation distance for different effects.

To derive more practical conclusions, a comprehensive analysis of various factors influencing the key rate of the protocol channel was conducted in this study. Figure 9a demonstrates that considering atmospheric turbulence alone results in a 28 dB loss at a propagation distance of 100 km. Integrating SDD, turbulence, weather visibility, and pointing errors increases this loss to about 60 dB at the same distance. Figure 9b illustrates that accounting solely for atmospheric turbulence allows our protocol to achieve nearly 100 km. However, incorporating weather effects reduces this distance by approximately 10 km, indicating a relatively minor impact on practicality. Yet, including SDD and pointing errors significantly reduces the maximum propagation distance to about 42 km. Thus, accounting for geometric attenuation is crucial for implementing the protocol. Furthermore, by combining the losses from Figure 9a and the propagation distances from Figure 9b (with a commonly accepted SKR threshold of $10^{-9}$), it is found that the farthest propagation distance depends on channel losses, identifying a maximum loss capacity of 26 dB for the UAV relay channel.

## 5. Conclusions

This paper introduced a transmission protocol for MDI-QKD that utilizes photon orbital angular momentum (OAM) encoding, with UAVs serving as relay platforms. By leveraging the mobility and maneuverability of UAVs, our protocol establishes a theoretically secure and efficient link, effectively mitigating potential threats posed by untrusted UAVs. The utilization of a photon OAM for information encoding mitigates the reference frame alignment issues, which are exacerbated by UAV platform jitter. To align more closely with practical applications, we conducted a comprehensive analysis of the impact of atmospheric turbulence, state-dependent diffraction (SDD), weather visibility, and pointing errors on free-space OAM-state transmission systems. This analysis elucidates the relationship between the key rate and propagation distance for our protocol. The results reveal that, under the identical setting conditions, considering SDD leads to a significant decrease in the key rate, equivalent to half of the previous data results. Furthermore, the study identifies the maximum channel loss capacity for this UAV relay platform as 26 dB. Thus, research will focus on overcoming various factors contributing to channel loss in the future, such as background light, APT system tracking errors, single-photon detection efficiency, and OAM sorter reception efficiency. This will provide theoretical support for specific experiments on UAV relay platform OAM-MDI-QKD, aiming to achieve a stable and efficient airborne quantum network.

## Figures and Tables

**Table 1:** Simulation parameters [38,39].

Parameter	Value	Parameter	Value
Dark count rate $P_{d}$ (counts/pulse)	$3\times 10^{-6}$	Link attenuation coefficient β (dB/km)	0.6
Altitude of UAV *h* (km)	10	Intensity of vacuum state $\mu_{0}\left(\nu_{0}\right)$	0
Beam radius $w_{0}$ (m)	0.02	Intensity of decoy states $\mu_{1}\left(\nu_{1}\right)$	0.10
Aperture diameter $D_{r}$ (m)	0.26	Intensity of signal states $\mu_{2}\left(\nu_{2}\right)$	0.36
Wavelength λ (nm)	1550	OAM topological charge *l*	4
Error correction efficiency *f*	1.16	Refractive-index structure parameter $C_{n}^{2}\;(m^{-2/3})$	$10^{-15}$
Finite size of date *N*	$10^{14}$	Standard deviation $n_{a}$	5
SUP basis misalignment error $e_{d}$	0.015	SPD efficiency $\eta_{d}$	0.6
Divergence of pointing jitter $\theta_{j}$ (μrad)	5		

## Data Availability

The data presented in this study are available on request from the corresponding author.
